# Fabrication of Chrysin-Loaded Hyaluronic Acid Decorated Niosomal Nanoparticles: Potential Anti-inflammatory and Anti-osteoclastic Effects on PBMCs of Rheumatoid Arthritis Patients

**DOI:** 10.34172/apb.43185

**Published:** 2025-01-05

**Authors:** Sarah Nadhim Sahib, Fadhil Jawad Al-Tu’ma, Atheer Hameed Odda, Maha M. Kadhim Al-Tu’ma

**Affiliations:** ^1^Department of Chemistry and Biochemistry, College of Medicine, University of Kerbala, Kerbala, Iraq.; ^2^Department of Medical Laboratories Techniques, College of Health and Medical Techniques, Al-Mustaqbal University, Babylon, Iraq.; ^3^Department of Anesthesia Techniques, College of Health and Medical Techniques, Al- Zahraa University for Women, Kerbala, Iraq.

**Keywords:** Rheumatoid arthritis, Chrysin, Inflammatory diseases, Niosome NPs, Hyaluronic acid

## Abstract

**Purpose:**

Rheumatoid arthritis is a persistent autoimmune condition characterized by joint inflammation and degradation, impacting individuals with varying degrees of severity. Chrysin is a natural flavonoid possessing diverse pharmacological properties and antioxidant and anti-inflammation activities. However, chrysin encounters limitations in bioavailability due to its low aqueous solubility and rapid metabolism. Targeted therapy using nanoparticle systems is a novel approach to overcome these difficulties.

**Methods:**

The hyaluronic acid-decorated niosomal nanoparticles (NPs) were fabricated using the thin-film hydration method and characterized by various techniques (DLS, AFM, SEM, FT-IR, and drug release pattern analysis). The peripheral blood mononuclear cells (PBMCs) were isolated from blood samples of patients with rheumatoid arthritis, and various factors levels, including nitric oxide, tumor necrosis factor alpha (TNF-α), interleukin (IL)-1β, IL-10, total antioxidative capacity (TAC), superoxide dismutase (SOD), glutathione peroxidase (GPx), as well as the expression levels of TIMP1, MMP9, and RANKL genes were evaluated.

**Results:**

The fabricated NPs demonstrated spherical morphology with 199±10.7 nm size, 0.653 PDI, and −15.38±2.8 zeta potential. The FT-IR results confirmed the successful incorporation of substances inside niosomal NPs. The treatment with chrysin loaded niosomal NPs successfully decreased the inflammatory agent (nitric oxide), inflammatory cytokines (IL-1β and TNF-α), and osteoclastic related genes (MMP9 and RANKL) expression level. On the other hand, the activity of antioxidant agents (TAC, SOD, and GPx), anti-inflammatory cytokine (IL-10), and anti-osteoclastic related genes (TIMP1) were found to increase.

**Conclusion:**

Taken together, the hyaluronic acid-decorated niosomal nano drug delivery system was acceptable in terms of characteristics and was able to direct the chrysin in the vicinity of PBMCs.

## Introduction

 Inflammatory diseases cover a range of conditions marked by long-term inflammation, which plays a role in developing and advancing several diseases.^1^ Inflammation is important for safeguarding organisms against substances and pathogens. It can also contribute to the progression of diverse diseases.^2^ This persistent inflammation leads to health issues such as cancer, diabetes mellitus, inflammatory bowel disease, obesity, rheumatoid arthritis, multiple sclerosis, osteoporosis, and neurological disorders.^3^ Pro-inflammatory cytokines are released when the inflammatory response is triggered by stimuli like toxic compounds (non-infectious substances), pathogens (viral or bacterial infections), and mechanical triggers (tissue injury).^4-6^ For instance, research has examined the connection between smoking and inflammatory bowel disease. There is evidence suggesting that tobacco usage might affect the progress of diseases.^7^

 Rheumatoid arthritis is a disease characterized by joint inflammation and damage. It affects patients with varying levels of severity.^8^ Rheumatoid arthritis characterized by levels of inflammation and oxidative stress which can lead to increased damage, to lipids, proteins, and DNA. This disease is associated with levels of stress and inflammatory markers along, with systemic complications, premature mortality and significant economic burdens.^8,9^

 The development of Rheumatoid arthritis involves a combination of factors, such as predisposition, exposure to triggers, and abnormalities in the immune system.^10^ This condition is characterized by increased stress and inflammatory markers leading to complications, premature mortality, and significant socioeconomic burdens.^11^ People with arthritis often experience a range of symptoms, including pain, fatigue, stiffness, and limited physical mobility. These symptoms significantly impact their quality of life.^12^ Moreover, rheumatoid arthritis is known to be a disorder that affects organ systems. It leads to deterioration and functional disabilities that can have outcomes.^13^ Researchers have studied the impact of arthritis on health conditions as well. For instance, individuals with arthritis have a risk of developing cardiovascular diseases. This highlights how the disease affects organs and functions within the body. Apart from joints, it may also affect organs like the heart, lungs, blood vessels, eyes, and skin. Rheumatoid arthritis is estimated to affect one in every two hundred individuals. Women are affected at rates compared to men, with a ratio of two to three times more cases in women. While it can occur in any age group; it commonly manifests between the ages of 50 and 59 years old.^14^ Individuals diagnosed with rheumatoid arthritis often exhibit a prevalence of risk factors associated with diseases such as obesity and dyslipidemia.^15^

 Early detection and accurate diagnosis are crucial for effective management, and disease-modifying agents, including biological agents, have significantly improved clinical outcomes.^16^ Over time, advancements have been made in the evaluation of features and understanding of the underlying mechanisms and therapeutic options for arthritis.^17^ Current management approaches for arthritis involve the utilization of tumor necrosis factor (TNF) inhibitors, methotrexate, and other targeted therapies.^18^ While traditional synthetic drugs used for treatment can have effects there is promising research on plants that possess anti-rheumatoid arthritis properties and show the potential in alleviating joint pain and inflammation.^19^

 Chrysin, a flavonoid, possesses pharmacological characteristics.^20^ Additionally, it demonstrates effects in terms of heart protection, antioxidant properties, neuroprotection, liver protection, anti-cancer properties, and potential use in diabetes treatment.^21^ Research has indicated that chrysin could be a candidate for treating arthritis due to its inflammatory and antioxidant effects.^22,23^ One of the challenges associated with chrysin is its low absorption by the body due to low solubility in water, rapid metabolism facilitated by UGTs and SULT enzymes, and efficient elimination through transporters like BCRP and MRP2.^24^ To address this issue, various formulations have been developed to enhance the bioavailability of chrysin.^25^ Niosomes are ionic surfactant vesicles that can encapsulate both hydrophilic and lipophilic pharmaceutical compounds. Their unique structure allows for controlled release profiles of drugs and improved stability and effectiveness.^26,27^

 Hyaluronic acid is a polymer with properties such as solubility, biocompatibility, and biodegradability. Through chemical modifications, it can be utilized as a drug delivery system with characteristics.^27^ This occurring polysaccharide is found within the body. It plays a role in tissues by being a part of the extracellular matrix. Its functions include regulating the interactions between growth factors, maintaining tissue volume, and providing lubrication.^28^ Hyaluronic acid has been studied for its potential applications in the treatment of inflammatory diseases, providing pain relief and exhibiting desirable biocompatibility and biodegradability.^29^ Hyaluronic acid can bind to CD44 receptors on immune cells in the dermal region, making it a potential carrier for site-specific dermal drug delivery in rheumatoid arthritis treatment.^30^

 In the current study, a hyaluronic acid-decorated niosomal nano drug delivery system was loaded with chrysin and occupied to target the peripheral blood mononuclear cells (PBMCs) derived from rheumatoid arthritis patients and evaluated different factors of these cells.

## Material and Methods

###  Nanoparticles synthesis

 Cholesterol (6 mg) and Span 60 (36 mg) were dissolved in methanol (6 mL) and chloroform (3 mL) and evaporated with a rotary evaporator at 120rpm at 60 °C for 1 hour to synthesize blank niosomal nanoparticles (blank Nio NPs). After the formation of the lipid-formed film, the temperature of the mixture was cooled to 24°C. The thin film was hydrated with phosphate-buffered saline (PBS) (10 mL) for 1 hour at 60 °C like the above. The final solution was mixed thoroughly by ultrasonication over an ice bath for 30 min in order to reduce the size of the synthesized NPs and stored at 4 °C. The chrysin loaded niosomal NPs (Nio-chr NPs) were synthesized with same method as above with addition of 2.54 mg chrysin to chloroform and methanol along with span 60 and cholesterol.

 To synthesized chrysin loaded hyaluronic acid coated niosomal NPs (H-Nio-chr NPs), 10 mL of normal saline containing 0.1% (w/v) hyaluronic acid solution was added dropwise to blank Nio-chr NPs, while the mixtures were stirring at ambient temperature for 1 h in order to reform the NPs and coating the hyaluronic acid on the NPs surface.

###  Morphology, size, and chemical interactions of NPs

 The size, poly dispersity index (PDI), and zeta potential of the synthesized niosomal NPs were analyzed by Zeta sizer dynamic light scattering system (ZS 90, Malvern Instruments Ltd., Malvern, UK). The surface morphological properties of the synthesized niosomal NPs were examined using scanning electron microscopy (SEM, MIRA3, TESCAN, Czech). Spectral analysis of the compounds before and after NPs preparation was analysed by using a Fourier-transform infrared (FT-IR) spectrophotometer (Shimadzu 8400 S, Kyoto, Japan) in the region of 4000-400 cm^1^ with spectra resolution of 4 cm^-1^.

###  Chrysin release from niosomal NPs

 To determine the in-vitro drug release profile of NPs dialysis membrane tube (12 kDa) was used. Briefly, 10 mL of H-Nio-chr NPs was transferred into a dialysis bag and placed in PBS (pH = 7.4) at 37 °C with gentle shaking at 100 rpm. At specific time intervals 5 mL of immersing buffer solution was analyzed with an ultraviolet spectrophotometry (PerkinElmer, Fremont, CA, USA) and replaced with fresh PBS. The absorbance of the immersed chrysin was measured at 367 nm (λmax of chrysin).

###  Study subjects

 The study protocol was approved by the Ethical Committee of the College of Medicine, University of Kerbala. Blood samples were obtained from healthy controls (n = 40), and rheumatoid arthritis patients (n = 35) who attended the orthopedics outpatient, Department of Rheumatology, Al-Hassan Teaching Hospital, Kerbala Health Directorate, Kerbala, Iraq with age range between October 2023 and January 2024. Patients were diagnosed based on the 2010 classification criteria for rheumatoid arthritis set by the European League Against Rheumatism (EULAR). Table 1 provides the clinical and demographical data of healthy controls and rheumatoid arthritis patients. Smokers, alcoholics, and patients suffering from chronic diseases or receiving non-steroidal anti-inflammatory drugs, disease-modifying antirheumatic drugs (DMARDs), and steroids were excluded from the study.

**Table 1:** Demographic and clinical data of healthy controls and patients with rheumatoid arthritis

	**Rheumatoid arthritis patients**	**Healthy controls**
Sex (Male/Female)	11/29	13/17
Body mass index (BMI)	24.91 ± 4.19	22.18 ± 2.07
ESR (mm/h)	58.12 ± 12.93	18.13 ± 7.83
DAS28 (28-Joint count disease activity score)	5.76 ± 0.94	-

###  PBMCs isolation and culture

 PBMCs were isolated by centrifugation over Histopaque 1077 (Sigma, Germany) density gradients. Then washed three times PBS and re-suspended in RPMI-1640 culture medium supplemented with 10% fetal bovine serum (FBS) (Biochrom, UK), 10 μg/mL of streptomycin (Sigma, Germany), and 10 U/mL of penicillin (Sigma, Germany).

###  Cell viability assay

 An MTT reduction assay determined the effect of various doses of free chrysin, Nio-chr NPs, and H-Nio-chr NPs on the viability of PBMCs. Firstly, 5 × 103 cells were seeded in each well of 96-well plates and incubated for 24 hours at 37 °C with 5% CO₂. The cells were treated with free chrysin (2.5-20 μM), Nio-chr NPs (2.5-20 μM), and H-Nio-chr NPs (2.5-20 μM) at 37 °C with 5% CO₂. After 48 hours, the medium containing treatment substances was replaced with 200 μL of MTT (Sigma, Germany) solution and incubated for 4 hours at 37 °C and in dark condition. The MTT solution was excluded from wells, and 200 μL of DMSO (Merck, Germany) was added to each well, followed by shaking on a plate shaker for 20 minutes. Finally, the optical density of wells was measured at 570 nm using the EL × 800 Microplate Absorbance Reader (Bio-Tek Instruments), and the cell viability effects of free chrysin, Nio-chr NPs, and H-Nio-chr NPs were calculated using GraphPad Prism 8.4 software.

###  Nitric oxide estimation

 The concentration of nitrite oxide in treated and untreated PBMCs supernatant was determined using measurement of residual nitrites by Griess’s method. PBMCs were seeded in 6-well plates (1 × 10^5^ cells), then incubated for 24 hours and treated with free chrysin, Nio-chr NPs, and H-Nio-chr NPs for 48 hours at 37 °C with 5% CO₂. Also, a group of cells received no substances as control. Afterwards, 100 μL of supernatants of PBMCs culture were incubated with the same amount of Griess reagent (Sigma, Germany) for 20 minutes at 24 °C in darkness. Finally, the absorbance at 450 nm was determined with a microplate absorbance reader (EL × 800, Bio-Tek Instruments), and the concentration of nitrite was calculated from a standard sodium nitrite (NaNO2) standard curve.

###  Anti-inflammatory and pro-inflammatory cytokine measurement

 PBMCs (1 × 10^5^) were seeded in a 6-well plate and incubated for 24 hours to attach the plates. Then PBMCs were treated with pure chrysin, Nio-chr NPs, and H-Nio-chr NPs for 48 hours at 37 °C with 5% CO₂. A group of cells remained untreated as a control. Finally, the IL-1β, TNF-α, and IL-10 levels in treated and untreated PBMCs were evaluated through an enzyme immunoassay using the human ELISA Kit (Sino Biological Inc., Beijing, China).

###  Determination of TAC, SOD, and GPx

 Total antioxidative capacity (TAC), superoxide dismutase (SOD), and glutathione peroxidase (GPx) levels in both treated and untreated PBMCs using the methodologies outlined by Erel,^31^ Marklund and Marklund,^32^ and Günzler and Flohé,^33^ respectively.

###  Real-time PCR

 Quantitative PCR analysis was conducted utilizing a LightCycler instrument (Roche Diagnostics). The amplification protocol involved an initial denaturation step at 95.8 °C for 10 minutes, followed by 40 cycles with the following conditions for the detection of MMP9, TIMP1, and RANKL: 95.8 °C for 5 seconds, primer annealing at 58.8 °C for 10 seconds, and primer extension at 72.8 °C for 20 seconds. Also, GAPDH expression was selected to normalize the expression levels of the intended mRNAs. Table 2 lists the primer sequences for quantitative PCR. Fluorescence emitted by SYBR Green I was detected at the conclusion of each amplification cycle to assess the accumulation of PCR products throughout the cycling process. Following each run, melting curve profiles were generated to validate the specificity of transcript amplification. The monitoring and quantification of fluorescence emission readings from cycle to cycle were conducted utilizing the second derivative maximum method through Light-Cycler Software. Standard curves for GAPDH and other primers were established by serially diluting total cDNA. All determined concentrations are expressed relative to the concentration of the respective standards.

**Table 2:** Primer sequences utilized for quantitative PCR

**Genes**	**Forward**	**Reverse**
MMP9	CCACTACTGTGCCTTTGAGTCC	AGAGAATCGCCAGTACTTCCC
TIMP1	CCTTCTGCAATTCCGACCTC	CATCTTGATCTCATAACGCTGGT
RANKL	GGATGGCTTTTATTACCTGT	AAAATTAACATTCAAAGGCAA
GAPDH	ATCCTGGGCTACACTGAGCAC	CCTGTTGCTGTAGCCAAATTCGT

## Results and Discussion

**Figure 1:**
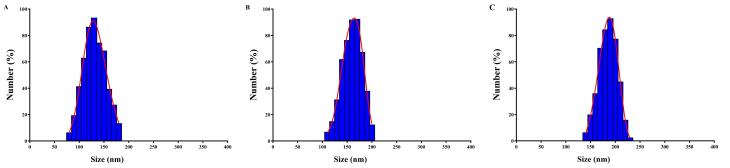
The DLS results of niosomal NPs A) blank Nio NPs, B) Nio-chr NPs, C) H-Nio-chr NPs. The size, zeta potential and PDI of fabricated NPs are in acceptable range

 Targeted therapy represents a personalized medicine approach that focuses on specific cells with minimal effect on healthy cells. It is based on the fact that these cells have specific molecular or genetic changes that distinguish them from normal cells.^34^ By targeting these changes, targeted therapies can selectively aim these cells to kill them, prevent their growth, and spread.^35^ For example, in cancer treatment unlike traditional chemotherapy, which can have widespread effects on the body and often causes side effects, targeted therapy drugs are designed to work more selectively and precisely, targeting specific molecules or pathways involved in cancer growth and progression.^36^ CD44 is an up-regulated receptor in rheumatoid arthritis that can be targeted for drug delivery strategies in rheumatoid arthritis therapy.^37^ It is a transmembrane glycoprotein that is targeted using hyaluronic acid in various drug delivery systems.^38^ For example, hyaluronic acid-decorated niosomal NPs have been used for targeted delivery of epirubicin to treat breast cancer.^39^ Niosomes are vesicular structures composed of nonionic surfactants and cholesterol that have been extensively studied for drug delivery applications.^40^ Niosomes are considered a promising carrier in advanced drug delivery, providing a controlled drug release system for an extended time period. Their biodegradability, non-toxicity, stability, and cost-effectiveness, make them distinguished compared to other NPs.^41^ Niosomes can be produced using different synthesis techniques, such as the thin film hydration method and the emulsification technique, allowing for large-scale production.^42^ In this study, the niosomal NPs were fabricated with the thin film hydration method. Figure 1 illustrates the DLS analysis of fabricated niosomal NPs. The mean diameter of blank Nio NPs, Nio-chr NPs, and H-Nio-chr NPs are estimated as 138 ± 14.1, 172 ± 8.4, and 199 ± 10.7 respectively. The H-Nio-chr NPs have the largest size compared to other NPs. This can be interpreted due to the loading of chrysin inside it and hyaluronic acid decoration on the surface of these NPs.

 Zeta potential is another essential surface parameter in the characterization of NPs. It is estimated the stability of nanomaterials and surface charge, as changes in these characteristics directly influence the biological activity of the NPs.^43^ Table 3 lists the zeta potential and the PDI value of niosomal NPs. Zeta potential value above + 30 to -30 mV prevents the aggregation of particles, which is crucial for maintaining the stability of the NPs.^44^ PDI provides information about the size distribution and uniformity of NPs. A low PDI value indicates a narrow size distribution, which is essential for ensuring the uniformity of nanoparticle performance, such as solubility, drug release, dissolution, and cellular uptake.^45,46^

**Table 3:** The size, zeta potential and PDI values for fabricated NPs

**Nanoparticles**	**Size (nm)**	**Zeta potential (mV) **	**PDI**
Blank Nio	138 ± 14.1	−12.74 ± 5.3	0.372
Nio-chr	172 ± 8.4	−18.76 ± 4.1	0.729
H-Nio-chr	199 ± 10.7	−15.38 ± 2.8	0.653

**Figure 2:**
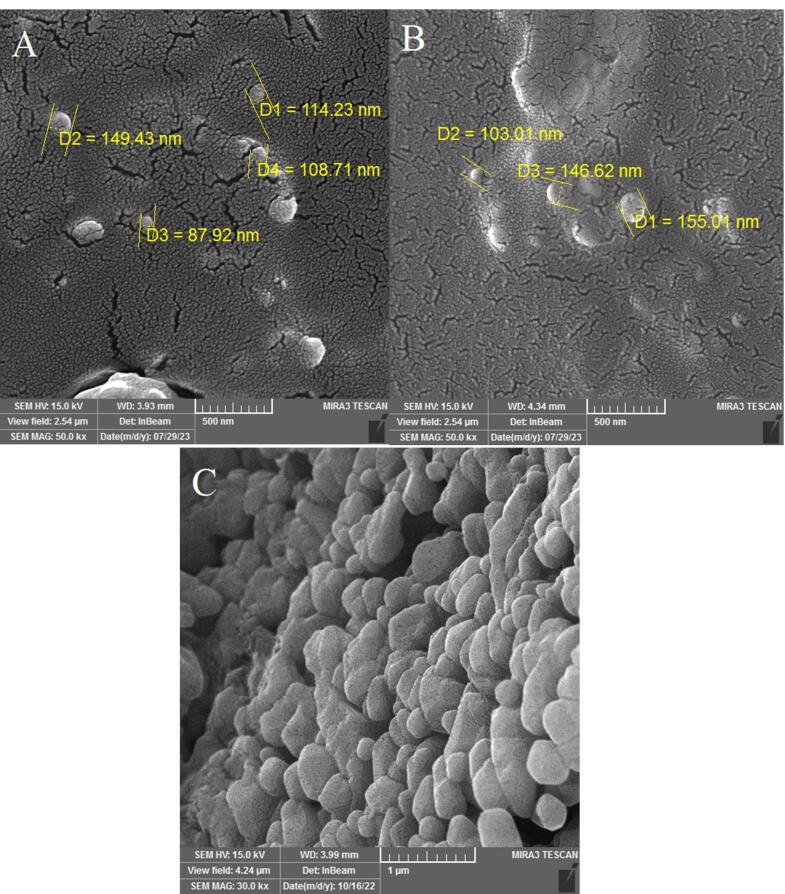
SEM images of niosomal NPs revealed their spherical morphology. A) blank Nio NPs, B) Nio-chr NPs, C) H-Nio-chr NPs

 Morphology is an effective factor in properties and potential applications of NPs. Studies have revealed that the shape of NPs can impact their circulation, distribution, extravasation, cellular uptake, and therapeutic performance.^47^ Previous studies collectively indicate that niosomes typically exhibit a spherical morphology.^48,49^ The SEM images of fabricated niosomal NPs show the same results (Figure 2).

**Figure 3:**
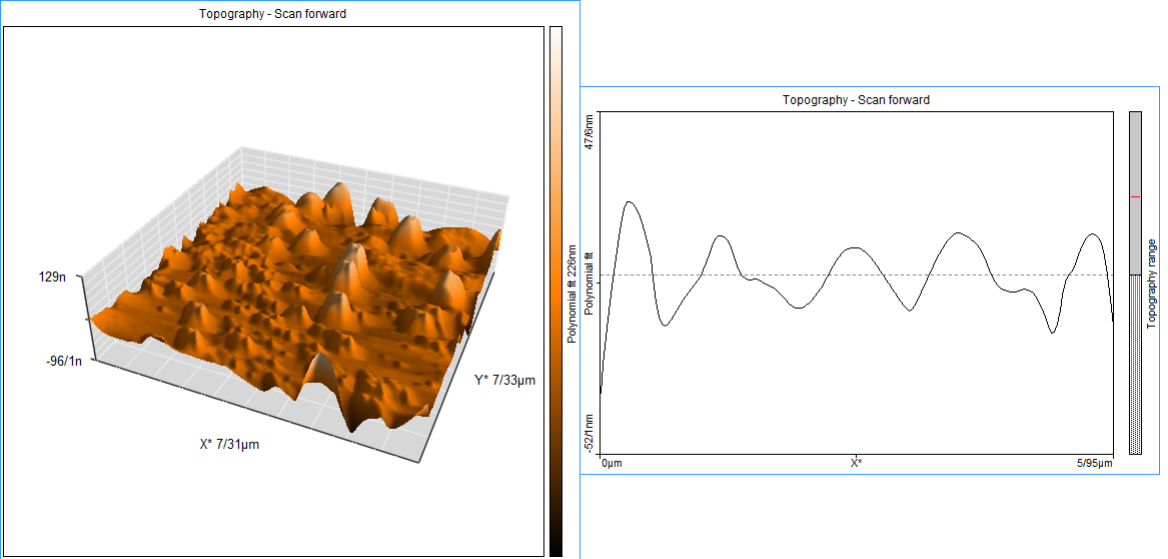
AFM image of Nio-chr NPs agrees with results of DLS and SEM images of H-Nio-chr NPs

 The results of AFM also showed the presence of particles with a maximum size of 129 nm, and the dispersion of NPs in a uniform manner and no aggregation of NPs are also evident in this image (Figure 3).

**Figure 4:**
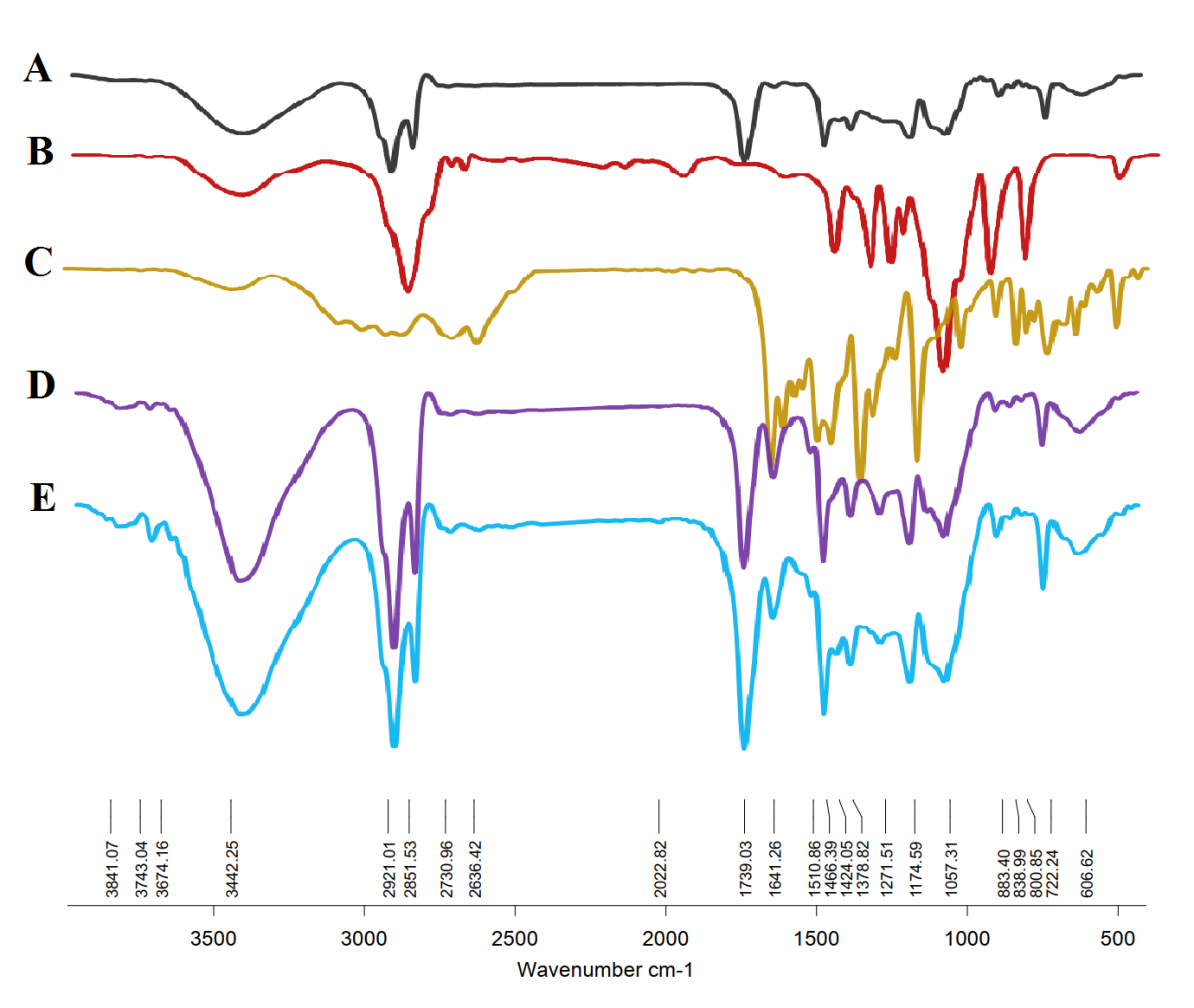
FTIR analysis of (A) blank Nio NPs, (B) hyaluronic acid, and (C) chrysin show their characteristic bands in FTIR of (D) Nio-chr NPs, and (E) H-Nio-chr NPs which confirms the successful incorporation of these substances into the final structure

 The confirmation of niosomal NP formation was achieved through FT-IR techniques. Chrysin manifested characteristic bands at 2625 cm^-1^ and 2343 cm^-1^, indicative of O–H stretching vibration and intramolecular hydrogen bonding.^50^ The FT-IR spectrum (Figure 4) of chrysin further revealed absorptions at 3012.79 cm^-1^ (OH), 2929.87 cm^-1^, 2713.84 cm^-1^, and 2630.91 cm^-1^ (C‒H stretching), and 1653.00 cm^-1^ (α, β-unsaturated carbonyl, C═O).^51^ These FT-IR results delineate peaks associated with functional groups inherent to the niosomal compounds, including the 1096 cm^-1^ peak linked to the stretching C–O alcohol bond in the structures of cholesterol and Span 60.^52^ The presence of a band at 1048.92 cm^-1^ is attributed to the C–O–C stretching vibration of hyaluronic acid.^53^ Upon the integration of hyaluronic acid into the drug-loaded niosome, a discernible peak at 1655 cm^-1^ corresponding to the amide group emerged, affirming the successful incorporation of HA into the final structure. The empty niosome displayed stretching peaks for C-O, C = O, and C-H at 1125 cm^-1^, 1747 cm^-1^, and 2900 cm^-1^, respectively. Furthermore, it manifested a carbonyl bond at 1625 cm^-1^ and a -NH stretching vibration at 3100–3400 cm^-1^, indicating of the successful formation of niosomes.^54^

**Figure 5:**
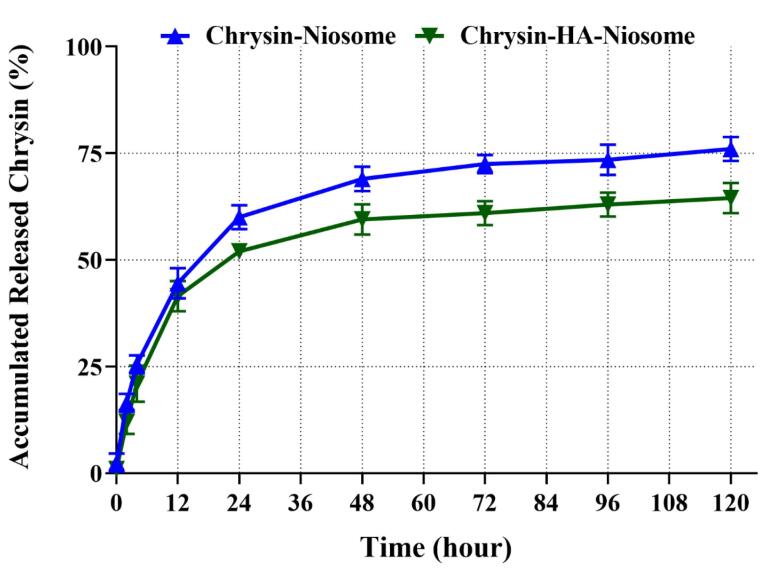
The 120 h invitro release experiment of chrysin from Nio-chr NPs, and H-Nio-chr NPs at 37°C and pH 7.4 show a biphasic release pattern

 The mechanism of drug release from NPs is influenced by various factors such as particle size, surface properties, and the porous structure of the NPs.^55-57^ A sustained drug release from NPs is considered desirable for medical applications.^58^ Niosomes are composed of biodegradable and non-immunogenic components that can carry both amphiphilic and lipophilic drugs, making them appealing for drug delivery.^59,60^ Niosomes have been reported to exhibit sustained release patterns for various drugs, such as α-tocopherol and dexamethasone, with cumulative release percentages ranging from less than 70% to an apparently biphasic release process.^61,62^ Figure 5 shows the 120 hours chrysin release pattern from Nio-chr NPs and H-Nio-chr NPs at 37 °C. After 120 hours, 64% and 76% of loaded chrysin were released from H-Nio-chr and Nio-chr NPs at pH 7.4, respectively. The observed release profile showed two distinct phases, with peak release rates of 37 and 43% in the initial 12 hours of the experiment, followed by a subsequent decline. This rapid initial release can be attributed to the surface attachment of the drugs to the niosomal NPs through weak bonds rather than encapsulation.

**Figure 6:**
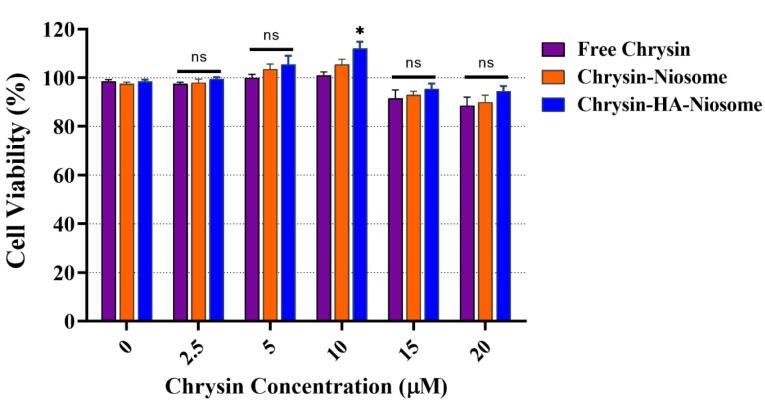
The proliferation effects of pure chrysin, Nio-chr NPs, and H-Nio-chr NPs on PBMCs

 The MTT reduction assay is a widely used method to measure cytotoxicity and cell viability. It is based on the conversion of MTT into formazan crystals by living cells, which is then quantified by measuring the absorbance at specific wavelengths.^63^ Figure 6 shows the inhibitory effect of pure chrysin, Nio-chr NPs, and H-Nio-chr NPs on PBMCs with various doses. Chrysin has been displayed to have a cytotoxic effect on cancer cells without affecting normal cells.^64^ The H-Nio-chr NPs composed of Span 60, cholesterol, chrysin, and hyaluronic acid. Hyaluronic acid and cholesterol are both natural components find in human body and studies confirmed their safety to normal cells.^65^ As illustrated in Figure 6, the free chrysin, Nio-chr NPs, and H-Nio-chr NPs have negligible and insignificant proliferation effects on PBMCs at 2.5, 5, 15, and 20 μM concentrations. The only significant result (*P < 0.1) is demonstrated in H-Nio-chr NPs treated group at 10 μM concentration, based on these results 10 μM of free chrysin, Nio-chr NPs, and H-Nio-chr NPs have been used for other experiments of this study.

**Figure 7:**
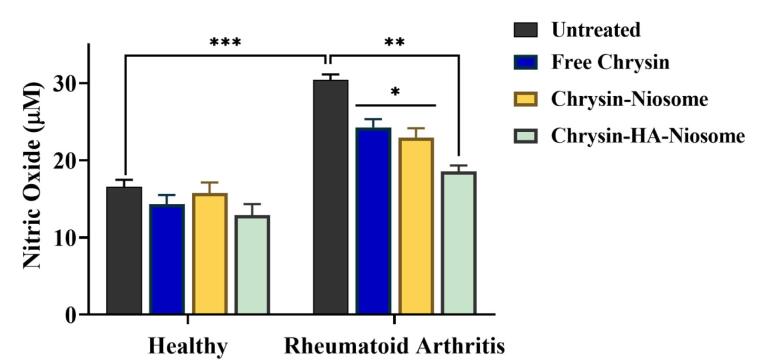
Nitric oxide level changes in untreated and pure chrysin, Nio-chr NPs, and H-Nio-chr NPs treated PBMCs

 Nitric oxide plays a significant role in the pathogenesis of rheumatoid arthritis.^66^ Excessive production of nitric oxide can lead to inflammation and contribute to the development of chronic inflammatory diseases, including rheumatoid arthritis.^67^ The nitric oxide/nitric oxide synthase signaling pathway is involved in the generation and release of inflammatory cytokines, oxidative stress, and joint damage in rheumatoid arthritis.^67^ Targeting nitric oxide synthase and its upstream and downstream signaling pathways may be an effective approach for managing rheumatoid arthritis.^68^ Furthermore, nitric oxide levels were found to be elevated in the serum of patients with rheumatoid arthritis compared to control group.^67^ Figure 7 shows the nitric oxide level of control (untreated) and pure chrysin, Nio-chr NPs, and H-Nio-chr NPs treated PBMCs. After 48 hours of treatment the nitric oxide level in pure chrysin, Nio-chr NPs, and H-Nio-chr NPs treated group was 24μM, 23μM, and 18μM, respectively. The better result of Nio-chr NPs group compared to pure chrysin can explain with niosome NPs ability to enhance bioavailability of chrysin, and the better result of H-Nio-chr NPs can explain with the targeted delivery of niosome NPs with hyaluronic acid.

**Figure 8:**
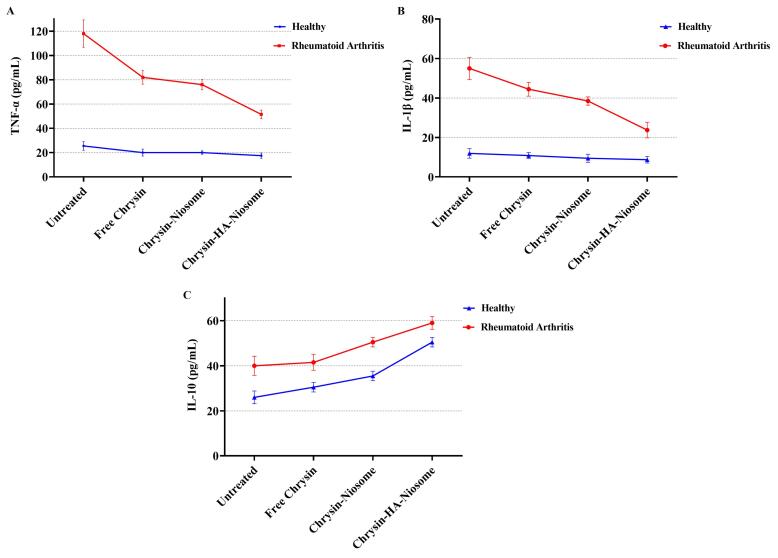
Comparison of (A) TNF-α, (B) IL-1β, and (C) IL-10 levels in untreated and treated (pure chrysin, Nio-chr NPs, and H-Nio-chr NPs) PBMCs

 TNF-α is a cytokine with proinflammatory properties that is involved in a wide range of physiological and pathophysiological functions. TNF-α has been implicated in autoimmune diseases, where clinically approved TNF-α inhibitors have shown potency in managing these conditions.^69^ In rheumatoid arthritis, TNF-α acts as a primary pathogenic driver, precipitating a pro-inflammatory cytokine cascade and tissue damage, and anti-TNF therapies have shown significant improvements in symptom scores.^70^ The IL-1 family of cytokines, including IL-1α, IL-1β, and IL-18, are associated with inflammation in rheumatic diseases, with IL-1β playing a pivotal role in promoting inflammation.^71^ IL-1β is an inflammatory cytokine that plays a major role in innate and adaptive immunity, particularly in driving inflammation and immune responses.^72^ IL-10 is a cytokine that plays a role in various diseases, including multiple sclerosis, cancer, and inflammatory diseases.^73^ The changes in IL-1β, TNF-α, and IL-10 levels in untreated and pure chrysin, Nio-chr NPs, and H-Nio-chr NPs treated PBMCs are shown in Figure 8, The highest reduction in TNF-α and IL-1β, levels compared to control group was achieved with H-Nio-chr NPs treatment. As previously described, this can be the result of hyaluronic acid coated on surface of niosome NPs which keeps them beside PBMCs and make cellular entrance easier for niosome NPs and chrysin. Unexpectedly there was an increase in the level of IL-10 in rheumatoid arthritis patients which were treated with pure chrysin, Nio-chr NPs, and H-Nio-chr NPs.

**Figure 9:**
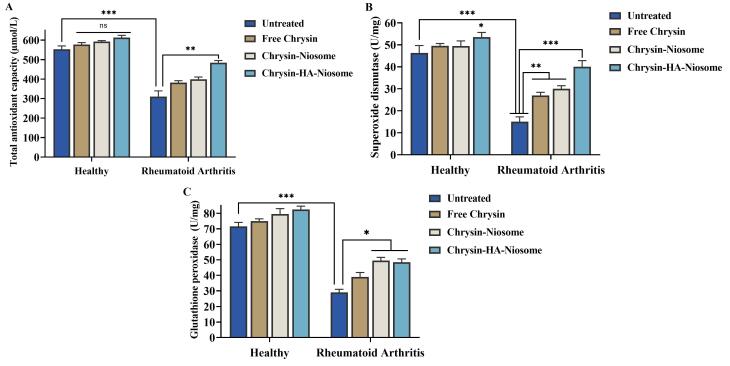
Comparison of changes in activity of(A) total antioxidant capacity, (B) superoxide dismutase, and (C) glutathione peroxidase in control and treated PBMCs

 TAC refers to the total antioxidant capacity, which is a measure of the ability of antioxidants to counteract oxidative stress and maintain redox balance in biological systems.^74^ One study found that participants in the top tertile of TAC were less likely to have rheumatoid arthritis, suggesting an inverse association between TAC and the risk of rheumatoid arthritis.^75^ Superoxide dismutase is an antioxidant enzyme that neutralizes superoxide radicals and protects against oxidative stress.^76^ It has therapeutic potential in rheumatoid arthritis by scavenging reactive oxygen species and mitigating inflammation.^77^ Glutathione peroxidase is an essential antioxidant enzyme that plays a significant role in protecting cells from oxidative damage by reducing hydrogen peroxide.^78^ Additionally, studies have indicated that decreased levels of reduced glutathione, an intracellular antioxidant, are associated with rheumatoid arthritis, further emphasizing the involvement of the glutathione defense system in the pathogenesis of the disease.^79,80^ As illustrated in Figure 9, the activities of TAC, GPx, and SOD were increased in treated PBMCs compared to untreated PBMCs.

**Figure 10:**
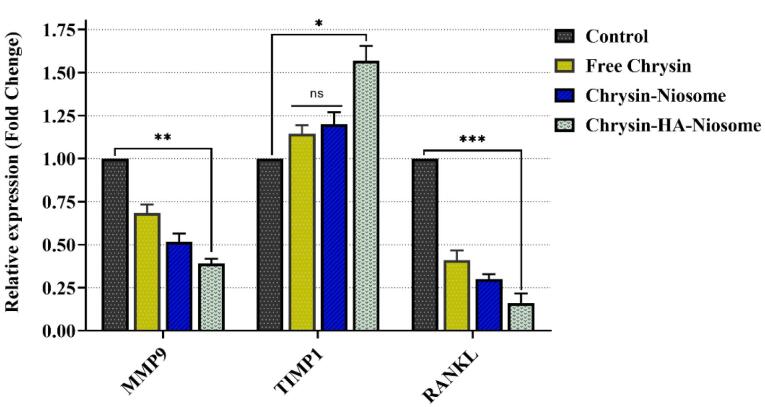
Expression level of MMP9, TIMP1, and RANKL genes in PBMCs before and after treatment with free chrysin, Nio-chr NPs, and H-Nio-chr NPs (*** *P* value < 0.001, ** *P* value < 0.01, and * *P* value < 0.1)

 A Real-time PCR was performed to investigate inflammation-related gene expression. Matrix metalloproteinase 2 (MMP2) plays an important role in rheumatoid arthritis progression, specifically in angiogenesis and invasion of tumor progression.^81^ The serum levels of MMP2 are significantly higher in RA patients compared to healthy group.^82^ MMP9 is associated with bone remodeling and is dysregulated in inflammatory diseases, including rheumatoid arthritis.^83^ The pathogenesis of chronic inflammation and arthritis is attributed to the production of MMP9 by macrophages in the tissue.^84^ RANKL (receptor activator of nuclear factor kappa B ligand) plays a critical role in osteoclast differentiation and bone destruction in rheumatoid arthritis.^85^ Studies have shown that RANKL is a key mediator of increased osteoclast activity in rheumatoid arthritis.^86^ Furthermore, increased RANKL activity has been revealed in diseases characterized by excessive bone loss, such as rheumatoid arthritis and osteoporosis.^87^ Figure 10 illustrates the expression level of these genes in PBMCs before and after treatment with pure chrysin, Nio-chr NPs, and H-Nio-chr NPs. The treatment with pure chrysin could downregulate the expression of MMP9 and RANKL while up-regulating the expression of the TIMP1 gene. It is evident that tissue inhibitor of metalloproteinases 1 (TIMP1) plays a crucial role in regulating the activity of MMP 2 and MMP9.^88^ As in previous tests, the best results were obtained with H-Nio-chr NPs.

 However, the TIMP1 expression change in pure chrysin treated and Nio-chr NPs group was not significant. This is the result of an enhancement in the bioavailability of chrysin on the one hand and, on the other hand, targeting and presence beside PBMCs with hyaluronic acid on the other hand.

## Conclusion

 In this study, the hyaluronic acid-decorated niosomal NPs were synthesized for the targeted delivery of chrysin. Their treatment demonstrated notable effects on PBMCs isolated from rheumatoid arthritis patients. Specifically, the hyaluronic acid-decorated niosomal NPs loaded with chrysin exhibited a significant reduction in nitric oxide levels (an inflammatory agent) and suppressed the activity of IL-1β and TNF-α (inflammatory cytokines), as well as expression of the MMP9, RANKL genes (osteoclastic related genes). Conversely, the treatment led to an increase in the activity of antioxidant agents like the TAC, SOD, GPx, IL-10, and anti-osteoclastic related gene (TIMP 2) expression. These findings collectively suggest the potential therapeutic efficacy of hyaluronic acid-decorated chrysin-loaded niosomal NPs in mitigating inflammation and modulating the immune response in rheumatoid arthritis patients. Further investigations, including in vivo studies and clinical trials, are warranted to validate and expand upon these encouraging results.

## Competing Interests

 No potential competing interest was reported by the authors.

## Data Availability Statement

 The data that support the findings of this study are available from the corresponding author, upon reasonable request.

## Ethical Approval

 This research protocol was evaluated and approved on 05.10.2023 by Ethical Committee of the College of Medicine, University of Kerbala.
